# Diagnostic accuracy of pattern differentiation algorithm based on Chinese medicine theory: a stochastic simulation study

**DOI:** 10.1186/1749-8546-4-24

**Published:** 2009-12-21

**Authors:** Arthur Sá Ferreira

**Affiliations:** 1 Department of Rehabilitation Science, Centro Universitário Augusto Motta, Av. Paris 72, Bonsucesso, Rio de Janeiro, BR CEP 21041-020, Brazil; 2 Department of Physical Therapy, Universidade Salgado de Oliveira, Rua Marechal Deodoro 263, Niterói, Rio de Janeiro, BR CEP 24030-060, Brazil

## Abstract

**Background:**

Clinical practice of Chinese medicine requires little information for differentiation of *Zang-fu *patterns. This study is to test the impact of information amount on the diagnostic accuracy of pattern differentiation algorithm (PDA) using stochastic simulation of cases.

**Methods:**

A dataset with 69 *Zang-fu *single patterns was used with manifestations according to the Four Examinations, namely inspection (Ip), auscultation and olfaction (AO), inquiry (Iq) and palpation (P). A variable quantity of available information (*N*_%_) was randomly sampled to generate 100 true positive and 100 true negative manifestation profiles per pattern to which PDA was applied. Four runs of simulations were used according to the Four Examinations: Ip, Ip+AO, Ip+AO+Iq and Ip+AO+Iq+P. The algorithm performed pattern differentiation by ranking a list of diagnostic hypotheses by the amount of explained information *F*_%_. Accuracy, sensitivity, specificity and negative and positive predictive values were calculated.

**Results:**

Use the Four Examinations resulted in the best accuracy with the smallest cutoff value (*N*_% _= 28.5%), followed by Ip+AO+Iq (33.5%), Ip+AO (51.5%) and Ip (52.0%). All tested combinations provided concave-shaped curves for accuracy, indicating an optimal value subject to *N*_%-*cutoff*_. Use of *N*_%-cutoff _as a secondary criterion resulted in 94.7% (94.3; 95.1) accuracy, 89.8% (89.1; 90.6) sensitivity, and 99.5% (99.3; 99.7) specificity with the Four Examinations.

**Conclusion:**

Pattern differentiation based on both explained and optimum available information (*F*_% _and *N*_%-*cutoff*_) is more accurate than using explained and available information without cutoff (*F*_% _and *N*_%_). Both *F*_% _and *N*_%-*cutoff *_should be used as PDA's objective criteria to perform *Zang-fu *single pattern differentiation.

## Background

In Chinese medicine, diagnosis often uses the Four Examinations (*Sizhen*), namely inspection (Ip, *wang*), auscultation and olfaction (AO, *wen*), inquiry (Iq, *wen*) and palpation (P, *qie*) [1-3]. In spite of ancient [4-8] and current [9,10] sources of extensive criteria for Chinese medicine diagnosis, studies on diagnostic strategies and objective criteria are inadequate [11-15]. Patterns, as related to illnesses in Western medicine, are composed of a set of signs and/or symptoms (i.e. manifestations) classified by Chinese medicine practitioners [10]. This set of manifestations is similar to a "cluster of symptoms" [16]. Although each pattern represents a broad description of its respective pattern - including onset, duration, location, progression and severity - those manifestations may not appear simultaneously [9,17]. Chinese medicine practitioners should be able to differentiate patterns based on minimal information.

### Computational approaches to Chinese medicine diagnosis

There are computational models for Chinese medicine pattern differentiation [1,3,14,18,19]. However, some of them were not described in detail and thus is difficult to compare results. Zheng and Wu [18] developed the Traditional Chinese Medicine Sizhen Integrated Recorder and Aided Syndrome Differentiator (TCM-SIRD) based on sensors (image, pulse and odor signal acquisition) and text information. No description was given on how the information was processed for diagnosis. No result regarding its diagnostic accuracy was reported. Yang *et al*. [19] developed the Information Management System of Traditional Chinese Medicine Syndrome Project based on Prior Knowledge Support Vector Machine (P-SVM), which uses the sequential minimal optimization procedure for training the classifier. They reported an accuracy rate of 95% with the trained P-SVM to classify a sample set of 2000 simulated records. No description of how the cases were simulated is available; thus, it is not possible to repeat the simulation procedure and to compare accuracy results. Huang and Chen [3] developed the Chinese Medical Diagnostic System (CMDS) for the digestive system. It uses a Web interface and expert system technology in diagnosing 50 types of digestive system diseases. The authors compared the diagnosis of 20 simulated cases made by CMDS and diagnosticians and found the results satisfactory; however, they did not report either simulation procedures or statistical validity. Wang *et al*. [1] designed a self-learning expert system with a novel hybrid learning algorithm GBPS* based on Bayesian networks. A dataset of 800 cases from real patients was used to train the Bayesian classifier. The maximum accuracy of 88% obtained for pattern differentiation was estimated by pseudo-random generation of a sample. Ferreira [14] proposed the pattern differentiation algorithm (PDA), whose objective criterion was based on pattern holism [10] because manifestations must be interpreted collectively rather than individually. This work simulated manifestation profiles from 69 *Zang-fu *single patterns and demonstrated the diagnostic accuracy to be 93.2% (sensitivity = 86.5%; specificity = 99.9%) can be obtained with PDA.

### Standard references for evaluating accuracy of pattern differentiation

Diagnosis established by expert Chinese medicine practitioners has been used as the standard for diagnostic accuracy tests of computational models [11-13]. However, the agreement in diagnosis among practitioners may be low (31.7%; 27.5-35%) [20], despite some improvement after training (73%; 64.3-85.7%) [21]. Standards for Reporting Interventions in Controlled Trials of Acupuncture (STRICTA) [22] recommend that the experience of Chinese medicine practitioners should be reported in clinical studies because such experience may influence diagnosis. As such, new diagnostic tests should not be used for comparison with diagnoses made by Chinese medicine practitioners but with methods that guarantee correct diagnosis.

Stochastic simulation models have been used for research in health sciences. A well-known simulation method is the Monte Carlo [23,24], in which the basic idea is to stochastically generate examples of a numerical variable and then evaluate the outcome of the model under evaluation. With stochastic methods, simulated patients can have their health status characterized by a computational model. For the determination of the accuracy of Chinese medicine diagnostic tests, a large number of patients with possible combinations of the manifestations for each pattern can be generated. The patterns must be differentiable; thus, it is virtually impossible to estimate the diagnostic accuracy without computer methods. However, some modifications based on Chinese medicine are needed to enable stochastic methods to process nominal variables.

### Objective criteria for Chinese medicine pattern differentiation

Recognition of factors related to the performance of diagnostic methods is relevant to the development of reliable methods that can be implemented for clinical and research purposes. For instance, the amount of information necessary to accurately perform pattern differentiation seems to be a key factor for Chinese medicine diagnosis [9]. Although Maciocia [9] stated that little information (i.e. few manifestations) is necessary for successful differentiation of *Zang-fu *single patterns, no evidence was presented to support this claim. Accurate diagnosis with minimum information is required to be recognized as "superior" traditional Chinese medicine practitioners, who detect patterns in early stages "to treat who is not yet ill" [4-6,25]. This statement suggests that patterns must be differentiated in early stages so treatment of unaffected systems can be initiated (according to the transmission effect). None of the abovementioned works [1,3,18,19] estimated the impact of available information on the accuracy of pattern differentiation.

This study aims to evaluate the effect of information amount on the diagnostic accuracy. The method was tested with *Zang-fu *single patterns using combinations of the Four Examinations of examination. It was hypothesized that the quantity of available information can optimally describe patterns, providing enough information for an accurate single pattern differentiation. Stochastic simulations and receiver operating characteristic (ROC) curves [26-28] were used to estimate the cutoff point of available information.

## Methods

The study was performed in the following sequence. First, computational simulation from patterns in a dataset was performed to obtain manifestation profiles that were applied to ROC curve analysis and the estimation of cutoff values for the available information. Next, the cutoff value for this new criterion was incorporated into PDA (as a secondary criterion to the explained information criterion), and the respective impact on the diagnostic accuracy was obtained with confusion matrices. All algorithms were implemented in LabVIEW 8.0 (National Instruments, USA) and executed on an 1.73 GHz Dual Core Intel^® ^microprocessor with 2.00 GB RAM running Windows Vista (Microsoft Corporation, USA). This work followed the Standards for Reporting of Diagnostic Accuracy [29] where applicable to simulation studies.

### Patterns dataset

The patterns dataset was developed in a previous work [14]. Sixty-nine *Zang-fu *single patterns (Additional file 1) [9] were listed, and all possible manifestations of each pattern *K *were listed separately according to the Four Examinations. The total quantity of manifestations describing pattern *K *in the dataset was represented by *N*_T,K_. Each entry in the dataset is separated by a comma and has case-insensitive letters. Manifestations were described as specifically as possible including onset ("palpitation in the morning," "palpitation in the evening"), duration ("acute headache," "chronic headache"), location ("occipital headache," "ocular headache") and severity ("dry tongue," "slight moist tongue," "moist tongue"), as well as any other characteristic that may be necessary to allow the pattern differentiation. Manifestations that co-occur in two or more patterns were assigned with the same term to increase the accuracy of string search algorithm. Patterns in the dataset have 16 (range 5-39) manifestations. A total of 504 manifestations were distributed among Ip (*n *= 108; 21%), AO (*n *= 36; 7%), Iq (*n *= 335; 66%), and P (*n *= 25; 6%).

Dataset consistency and quality were computationally tested before the simulation and diagnostic procedures. Internal (intrapattern) and external (interpattern) exploratory analyses were performed with string search algorithm. Intrapattern consistency was obtained by excluding repetitions of any manifestation in the same examination method, as well as among the Four Examinations describing the respective pattern. Interpattern consistency was obtained by ensuring that two patterns were not described with the same complete manifestation profile (both constitute the same pattern). Patterns in the dataset are mutually exclusive and collectively exhaustive, that is, for each manifestation there is at least one possible pattern, and there is no pattern without manifestations (considering the Four Examinations).

### Manifestation profile simulation algorithm

#### Study population

Cases (true positive) and controls (true negative) manifestation profiles were generated by the manifestation profile simulation algorithm (MPSA). The inclusion criterion was the simulation of cases representing a *Zang-fu *single pattern in the dataset. For both types of simulation, it was assumed that the probability of each manifestation in the general population is given and follows a uniform distribution.

#### Sample size

There is no formula specifying the exact number of simulations needed in stochastic simulation studies, but the number should increase with the complexity of the patterns to reduce simulation variability in the result [30]. Thus, the sample sizes were estimated based on the previous results of PDA [14] and equations (1) to (4), which were derived for detecting differences in accuracy tests using ROC curves [26]:(1)

Where *AUC*_*i *_is the area under the ROC curve calculated for each new criterion *N*_% _(*i *= 1) and *N*_%-*cutoff *_(*i *= 2).

A sample size of at least 5734 manifestation profiles (84 true positive/pattern and 84 true negative/pattern, summing to 11,468 cases/examination method) is necessary to detect a 1% difference in accuracy (best accuracy obtained with PDA in the previous work = 93.2%) [14], with *α *= 5% (*Z*_*α *_= 1.645, one-sided test significance) and *β *= 90% (*Z*_*β *_= 1.28, power of test).

#### Participant recruitment and sampling

Four runs of simulations were performed according to the following combination of examination methods: Ip, Ip+AO, Ip+AO+Iq and Ip+AO+Iq+P. Two hundred manifestation profiles (100 true positive and 100 true negative cases) were prospectively generated for each of the 69 patterns, summing to 13,800 cases per examination method. Globally, 55,200 cases were simulated and tested.

#### Data collection (simulation) of true positive cases

True positive cases of *Zang-fu *pattern *K *were simulated by selecting from the dataset a random quantity (*N*_*R*, *K*_) in the interval (1;*N*_*T*, *K*_), according to the selected methods of examination. Each sorted manifestation was excluded from the set of possible manifestations to prevent multiple occurrences of the same manifestation at the respective simulated case. This iterative process continued until the *N*_*R*, *K *_manifestations were sorted to generate the manifestation profile.

#### Data collection (simulation) of true negative cases

To obtain a true negative case for the same pattern *K*, this respective pattern was removed from the dataset and the same quantity *N*_*R*, *K *_(from the total of manifestations for the excluded counterpart true positive pattern, *N*_*T*, *K*_) was selected from the entire dataset and respective examination methods. In other words, the MPSA sorted *N*_*R*, *K *_manifestations from the entire dataset after the exclusion of pattern *K*. This procedure allows a quantitative pair-wise comparison between true positive and true negative cases with respect to the available information for pattern *K*, *N*_%, *K*_. Although the true positive pattern was removed from the dataset, its manifestations that co-occur in other patterns are still present and could be selected to compose a true negative manifestation profile.

#### Missing cases

Because patterns may not present manifestations for some of the examination methods, empty manifestation profiles related to these examination methods represent missing cases and were excluded from analysis.

#### Reference standard

Because cases were simulated from all possible manifestations of each pattern in the dataset, the output of the diagnostic algorithm was compared to the actual name of the simulated pattern in the dataset. Thus, it was considered a gold-standard method. The result was treated as a binary variable for further classification according to the confusion matrix displayed in Table 1.

**Table 1:** Confusion matrix for assessment of diagnostic accuracy between the reference test and pattern differentiation algorithm

		Simulation test result (gold-standard)	
		
		Simulation pattern	Other patterns
Prediction test result	Identified pattern	TP †	FP ‡
	Other patterns	FN ‡	TN †

TP: true positive, TN: true negative, †: successful pattern differentiation, ‡: unsuccessful pattern differentiation

Calculations displayed in this table are related to equations (8) to (12).

All simulated cases were evaluated by PDA. No user intervention was required during the entire process (simulation; identification with *F*_%_; cutoff estimation for *N*_%_; identification with *F*_%_, *N*_%_, and *N*_%-*cutoff*_; and statistical analysis). Additionally, MPSA and PDA are composed of independent algorithmic codes (i.e., there is no code sharing), so the results of the identification were considered to be blinded to the simulation parameters.

### Pattern differentiation algorithm (PDA)

#### Strategy description

The first version of PDA was developed and validated previously for *Zang-fu *single patterns [14]. Its strategy was based on the reasoning that as more manifestations are explained by a single pattern, the higher the probability of this respective pattern to be the diagnosis. An implicit assumption is that the patients are capable of reporting their symptoms and that the Chinese medicine practitioners are able to correctly identify manifestations. Briefly, the algorithm performed pattern differentiation in a three-stage schema using the same pattern dataset used for simulation of true positive and true negative cases. Below is presented the pseudocodes of PDA algorithm:

**Step 1. Initialize vectors**. CP = [], DH = [], F_%,K _= [], N_%,K _= []

**Step 2. Input simulated (or real) data**. M = [m_1_, m_2_,..., m_Np_]

**Step 3. Calculate explained (F_%,K_) and available (N_%,K_) information for pattern K on dataset**. F_%,K _= N_E,K_/N_P _× 100%; N_%,K _= N_E,K_/N_T,K _× 100%

Step 4. Populate vectors CP, F_%,K_, and N_%,K_

Step 5. Populate diagnostic hypothesis (DH) from CP with patterns in which F_%_>0%. Filter F_%,K _and N_%,K_ accordingly

Step 6. Diagnosis and output

6.1 Check for the existence of diagnostic hypotheses

size(DH) = 0 ⇒ display("No *Zang-fu *single pattern found");

6.2. Check for the existence of a single diagnostic hypothesis

size(DH) = 1 ⇒ display("Diagnosis = DH(1)");

6.3. Differentiate patterns between two or more diagnostic hypothesis

sort DH, F_%,K_, and N_%,K _by descending values of F_%,K _and ascending values of N_%,K _simultaneously

if F_%,K_(1)>F_%,K_(2) AND N_%,K_(1)<N_%,K_(2) ⇒ display("Diagnosis = DH(1)");

else ⇒ display("*Zang-fu *single pattern differentiation was not possible with these manifestations").

#### Data entry and hypotheses generation

The first stage comprised data collection and a dataset search. During data entry of manifestations, PDA searched with a combinatorial procedure for quoted terms to increase the sensitivity of the method. Sequentially, a list of candidate patterns was generated with patterns that explain at least one manifestation collected at the exam. Patterns with no manifestations recognized were not used for further analysis.

#### Ranking hypotheses by the quantity of explained information

The candidate patterns were ranked by the amount of explained information, *F*_% _(equation 5):(5)

Where *N*_*E*, *K *_is the number of explained manifestations for pattern *K *in the diagnostic hypothesis list and *N*_*P *_is the number of presented manifestations either from simulated profiles or real patients.

Thus, *F*_%, *K *_represents the explained information of pattern *K *on the clinical history. Notice that for the simulated true positive and true negative profiles *N*_*R*, *K *_= *N*_*P*_. It is expected that the occurrence of a successful pattern differentiation increases with decreasing *F*_%, *K*_. Hence, the candidate patterns ranked in descending order of *F*_% _represent diagnostic hypotheses.

#### Pattern differentiation

In this last stage, the differentiation was considered successful if PDA found a single pattern *K *among diagnostic hypotheses with the highest, unique *F*_%_. Otherwise, if at least two (or several) patterns were found among diagnostic hypotheses with high, equal values of *F*_%_, the procedure was unsuccessful because differentiation among single patterns was not possible with the explained information.

### Calculation of the additional criterion: available information

Patterns maybe described by different quantities of manifestations. As such, the available information must be normalized to allow comparison among them. The normalized available information was calculated by equation 6:(6)

Where *N*_*E*, *K *_is the quantity of explained manifestations for pattern *K*. Note that for simulated true positive profiles *N*_*E*, *K *_= *N*_*R*, *K *_because all sorted manifestations will be explained by pattern *K*. It is expected that the occurrence of a successful pattern differentiation increases with increasing *N*_%, *K*_.

### Statistical analysis

#### Outputs from algorithms for statistical analysis

The MPSA output for each manifestation profile: binary classification of the simulated case as true positive (= 1) or true negative (= 0); the name of the simulated pattern *K*;*N*_*R*, *K*_; *N*_*T*, *K *_and the manifestations as quoted terms, words separated by commas. PDA received the manifestation profile from MPSA and output for each tested profile: the name of the identified pattern (the diagnosis);*F*_%, *K*_*N*_%, *K *_and the quantity of candidate patterns *N*_*CP*_. The diagnosis of each manifestation profile was made according to the respective method used for the simulation of cases (e.g. profiles simulated with Ip were diagnosed using only Ip, and so on).

#### Estimation of cutoff for the available information

An algorithm for estimation of the optimum quantity of available information was implemented and integrated into MPSA and PDA. To determine the degree of available information that maximizes PDA's accuracy, its binary output to true positive cases was used as the reference method for evaluation of the new test. *N*_% _was used as the prediction variable to generate confusion matrices for each simulation run according to the examination methods. Cutoff values for *N*_% _were evaluated by maximizing both sensitivity and specificity obtained with ROC curves [26-28]. The smallest cutoff value was the minimum observed test value minus 1, and the largest cutoff value was the maximum observed test value plus 1. All the other tested cutoff values were the averages of two consecutive ordered observed test values.

#### Estimation of the diagnostic accuracy of PDA with both explained and available information criteria

The cutoff values of *N*_% _obtained for each combination of examination methods were incorporated as the second criterion for pattern differentiation. Because of the nature of ROC curve analysis [26], the accuracy of a diagnostic test is expected to decrease with either lower or higher cutoff values - producing a concave-shaped curve. Therefore, it is appropriate to subtract the cutoff values from *N*_% _to dislocate the accuracy curve to its optimum operating point (equation 7):(7)

Where *cutoff *represents the point of maximum accuracy obtained with each examination method. As such, the maximum accuracy is associated with the minimum *N*_%_. The identified pattern is then chosen by simultaneously arranging the diagnostic hypotheses by descending values of *F*_% _and by ascending values of *N*_% _or *N*_%-*cutoff*_.

#### Comparison between PDA with *N*_% _and *N*_%-*cutoff*_

Both conditions (*N*_% _and *N*_%-*cutoff*_) were tested to evaluate the effect of curve dislocation on diagnostic accuracy. All results were organized according to the examination methods. PDA was evaluated with estimations of accuracy (proportion of true results in the population, equation 8), sensitivity [31] (proportion of successful pattern differentiations that are correctly predicted by PDA, equation 9) and specificity [31] (proportion of unsuccessful pattern differentiations that are correctly predicted by PDA, equation 10) and negative and positive predictive values [32] (equations 11 and 12 respectively) (Table 1).(8)

Where *TP *and *TN *represent the number of true positive and true negative cases respectively; *FP *and *FN *represent the number of false positive and negative cases respectively.

The 95% confidence interval (95%*CI*) for the above binomial proportions *p *(equations 8-12) was calculated with Wilson's method (equation 13) [33]:(13)

Where *p *is the binomial proportion, *Np *is the sample size of the binomial proportion *p*, and *Z*_*α*/2 _is the two-sided significance value (*α*_5% _= 1.96). As the tests (with both *N*_% _and *N*_%-*cutoff*_) have been evaluated on the same samples of true positive and true negative cases, an adaptation of McNemar's test for correlated proportions is applied [24]. The true positive and the true negative subjects are divided into four parts according to their test responses (Table 2). Hence, for comparison of the results of the two tests with equal sample sizes (*k *= *TP*/*TN *= 1) the test statistic is (equation 14):(14)

**Table 2:** Confusion matrices for comparison of binomial proportions between the two diagnostic tests

	**TN (no single pattern simulated)**				**TP (single pattern simulated)**		
			
	**Test 1**	**-**	**+**		**Test 1**	**+**	**-**
	
**Test 2**	-	A	B	**Test 2**	+	a	b
	+	C	D		-	c	d

TN: true negative, TP: true positive

(+) and (-) indicate positive and negative test results respectively.

B is the number of TN cases classified correctly by test 2 and falsely by test 1 and conversely for C cases and analogously for the TP group.

Where B is the number of true negative cases classified correctly by *N*_%-*cutoff *_and falsely by *N*_% _and conversely for C cases and analogously for the true positive group.

Estimations related to ROC (*AUC *and respective95%*CI*) were obtained with the nonparametric Wilcoxon statistic [26,27] (equations 15-20):(15)

Where *N*_%, *S *_and *N*_%, *U *_stand for the quantity of cases with successful and unsuccessful procedures at *N*_% _respectively; *n*_S _and *n*_*U *_stand for the sample sizes of successful and unsuccessful procedures; *Q*_1 _represents the probability that two randomly chosen unsuccessful procedures will both be ranked with greater suspicion than a randomly chosen successful procedure; and *Q*_2 _represent the probability that one randomly chosen unsuccessful procedure will be ranked with greater suspicion than two randomly chosen successful procedures.

#### Test reproducibility

Calculations of reference standard reproducibility were not performed because both true positive and true negative cases were always generated from the dataset.

## Results

**Figure 1:**
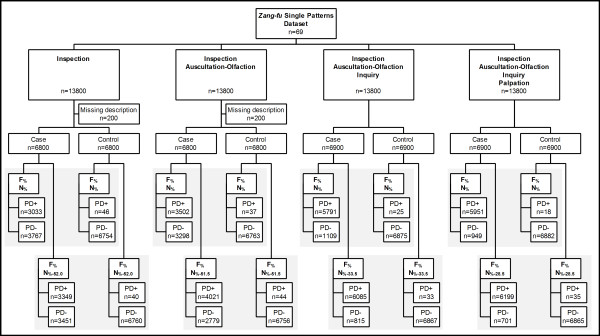
**Flowchart describing the simulation study**. Departing from *Zang-fu *single patterns dataset, manifestation profiles were simulated according to the combination of traditional examination methods. Cases (true positive) and controls (true negative) profiles were tested with criteria *F*_% _and *N*_% _and with *F*_% _and *N*_%-*cutoff *_for comparison of diagnostic tests. PD+: successful pattern differentiation; PD-: unsuccessful pattern differentiation.

The flowchart describing this simulation study is presented in Figure 1. Regarding the simulation process, 200 of 13,800 (1.4%) cases were excluded from both Ip and Ip+AO examination methods due to the absence of manifestations in those respective examination methods in the dataset.

### Example

Consider the heart-yang collapse pattern (Additional file 1, *K *= 35) described in the pattern dataset by the following manifestations arranged by examination methods: Ip35: cyanosis of the lips, comma, pale tongue, blue-purpur tongue, short tongue; AO35: weak and shallow breathing, dyspnea; Iq35: frequent palpitation, profuse sweating; and P35: cold limbs, knotted pulse, minute pulse, hidden pulse. The MPSA sorted 4 (= *N*_*R*,35 _= *N*_*P*_) manifestations within the Four Examinations and obtained the following manifestations: cyanosis of the lips, comma, frequent palpitation, knotted pulse. This manifestation profile resulted in: *N_CP_* = 5; diagnostic hypotheses = (heart-yang collapse; liver wind agitating within, extreme heat generating wind; heart-blood stasis; heart-yang deficiency; phlegm-fire harassing the heart); *N*_*E *_= (4; 1; 2; 1; 1); *N*_*T *_= (13; 14; 12; 18; 30); *F*_% _= (100; 25; 50; 25; 25); *N*_% _= (30.8; 7.1; 16.7; 5.6; 3.3). After ranking the diagnostic hypotheses by *F*_%_, the pattern "heart-yang collapse" was successfully identified as the diagnosis (binary classification = 1) because that pattern explained 100% (*F*_%_) of the presented manifestations. The available information for diagnosis in this case of "heart-yang collapse" was *N*_% _= 30.8%.

### MPSA and PDA algorithms performance

**Figure 2:**
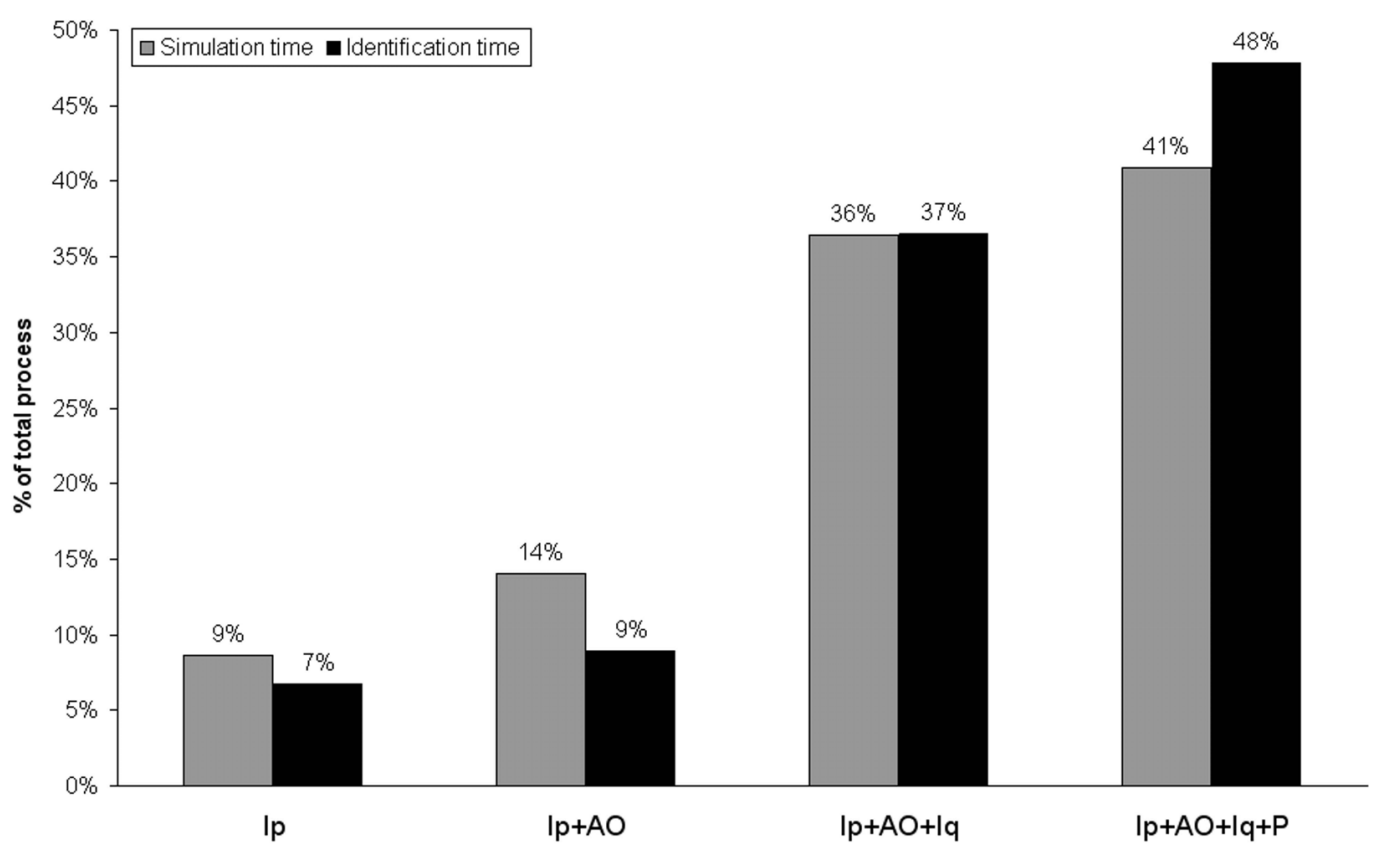
**Percent time interval for execution of the simulation (MPSA) and identification (PDA) algorithms**. The combination of methods progressively increased the duration of both simulation and identification processes.

Figure 2 summarizes the percent time interval spent in simulation (by MPSA) and diagnosis (by PDA). Simulation of 13,800 cases lasted 3.5s (Ip), 5.7s (Ip+AO), 14.8s (Ip+AO+Iq), and 16.6s (Ip+AO+Iq+P), with a total simulation time of 40.6s. Diagnosis of the simulated cases lasted 3 minutes and 30s (Ip), 4 minutes and 38s (Ip+AO), 18 minutes and 57s (Ip+AO+Iq), and 24 minutes and 49s (Ip+AO+Iq+P), with a total simulation time of 51 minutes and 54s. From these results, the average time for diagnosis of each case with the Four Examinations is estimated in less than 0.1s.

### ROC curves and cutoff values for N_%_

**Figure 3:**
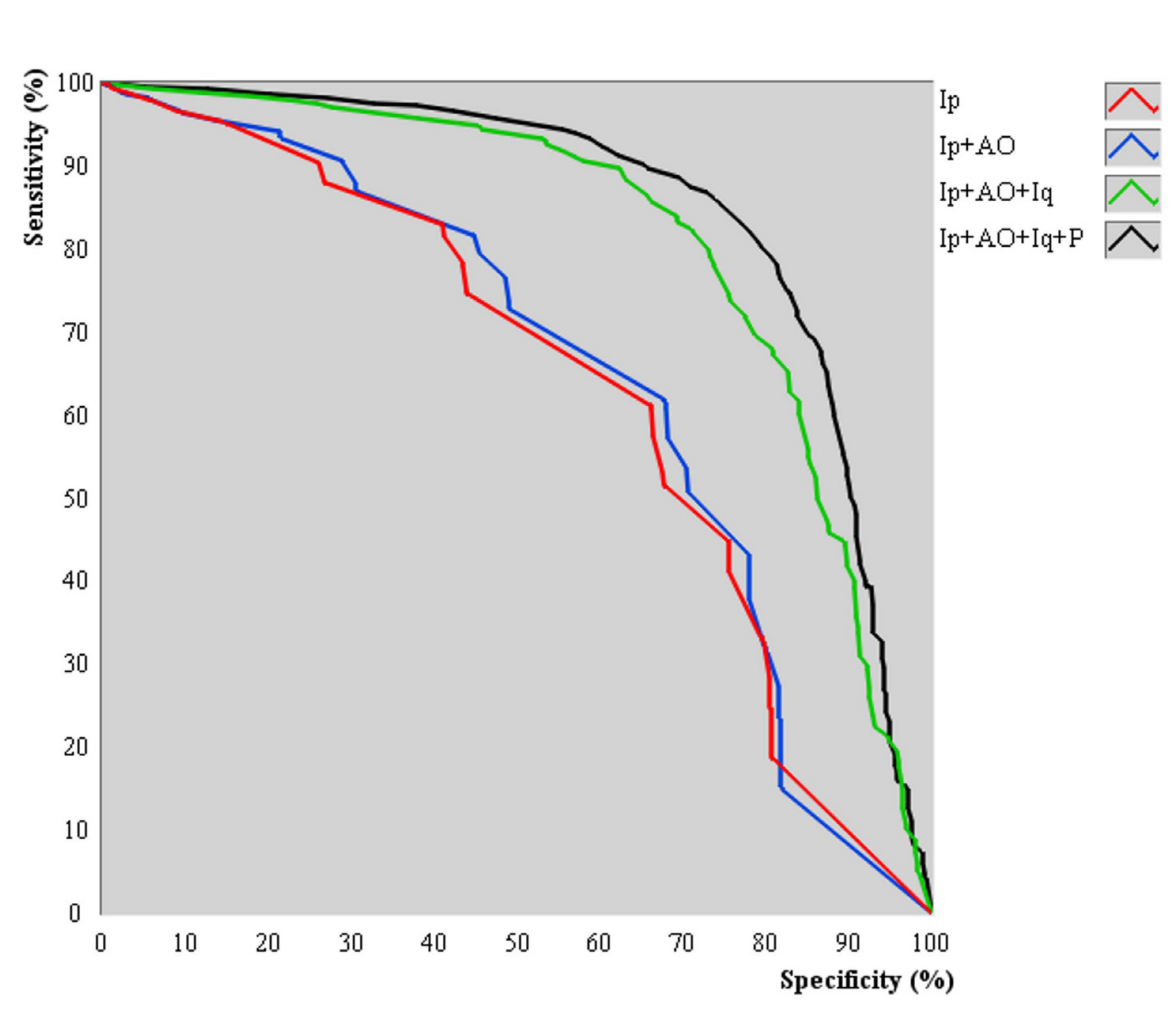
**Receiver operating characteristic curves with respect to the available information for diagnosis**. The combination of methods yielded distinct cutoff points. The Four Examinations (Ip+AO+Iq+P) resulted in the best overall statistical performance with the minimum cutoff value of available information, followed by three (Ip+AO+Iq), two (Ip+AO) and single (Ip) examination methods.

ROC curves obtained for *N*_% _grouped by the examination methods are shown in Figure 3. The respective values of cutoff, accuracy, *AUC*, sensitivity and specificity are presented in Table 3. The combination of Examinations methods yielded distinct cutoff points to be used by PDA. The use of the Four Examinations resulted in the best overall statistical performance with the minimum cutoff value of available information (*N*_% _= 28.5%), followed by three (Ip+AO+Iq, *N*_% _= 33.5%), two (Ip+AO, *N*_% _= 51.5%) and single examination methods (Ip, *N*_% _= 52.0%).

**Table 3:** Cutoff values of available information and related statistical measures according to the examinations

	Cutoff for criterion: N%			
	
Methods	Ip	Ip+AO	Ip+AO+Iq	Ip+AO+Iq+P (Four Examinations)
**N simulated**	13800	13800	13800	13800
N missing*	200	200	0	0
**Cutoff**	52.0%	51.5%	33.5%	28.5%
**Accuracy**	64.0% (62.9; 65.2)	64.9% (63.8; 66.1)	74.7% (73.7; 75.7)	80.1% (79.2; 81.1)
**Area under the curve**	63.4% (62.0; 64.7)	64.6% (63.3; 65.9)	81.6% (80.1; 83.1)	85.3% (83.8; 86.8)
**Sensitivity**	61.3% (59.6; 63.2)	61.9% (60.3; 63.6)	74.5% (73.4; 75.7)	80.2% (79.2; 81.2)
**Specificity**	65.9% (64.5; 67.4)	67.7% (66.2; 69.3)	75.5% (73.2; 78.1)	79.5% (77.3; 82.2)

Ip: inspection, AO: auscultation-olfaction, Iq: inquiry, P: palpation

Note:Missing cases are due to absence of manifestations describing the inspection method.

**Figure 4:**
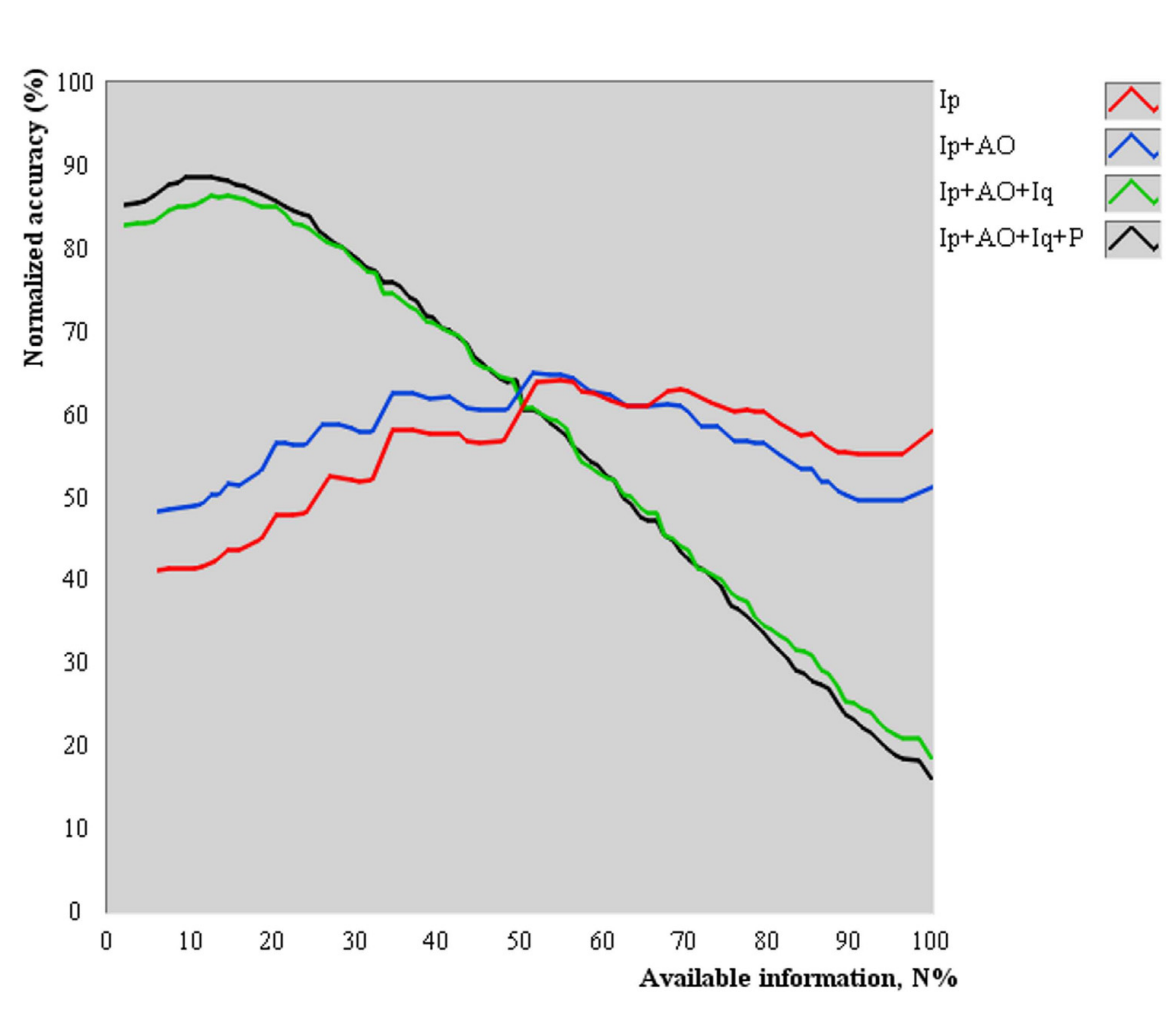
**Average normalized accuracy as a function of the available information for diagnosis**. All tested combinations provided concave-shaped curves, indicating that there is an optimum quantity of available information to accurately perform pattern differentiation. Also, inspection (Ip) or its combination with auscultation-olfaction (Ip+AO) provided lower average normalized accuracies than addition of inquiry (Ip+AO+Iq) and palpation (Ip+AO+Iq +P; Four Examinations) to the examination, as indicated by the relative position of the concavity (maximum value).

The average accuracy of PDA as a function of *N*_% _is shown in Figure 4. All tested combinations provided concave-shaped curves, indicating that there is an optimum quantity of available information to accurately perform pattern differentiation. Also, inspection (Ip) or its combination with auscultation-olfaction (Ip+AO) provided lower average accuracies than the addition of inquiry (Ip+AO+Iq) and palpation (Ip+AO+Iq+P) to the examination procedure, as indicated by the relative position of the concavity (maximum value).

**Figure 5:**
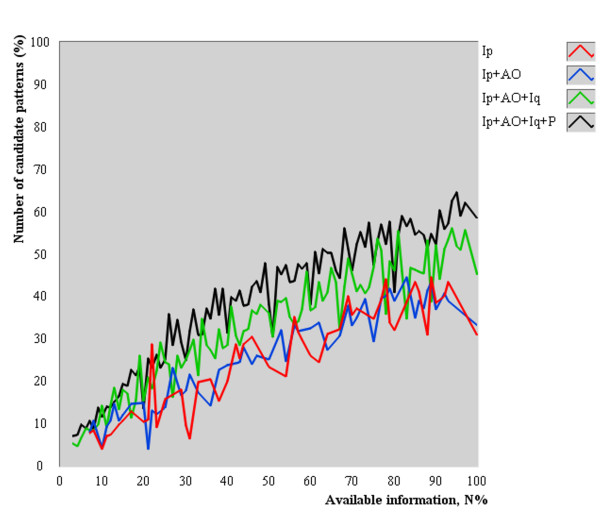
**Average number of candidate patterns as a function of available information for diagnosis**. The average normalized number of candidate patterns increased as more information was available to perform pattern differentiation.

The *N*_*CP *_as a function of *N*_% _is shown in Figure 5. *N*_*CP *_was normalized to the quantity of patterns in dataset. The average normalized *N*_*CP *_increased as more information was available to perform pattern differentiation.

### Comparison of diagnostic accuracy of PDA with both explained and available information (N_% _and N_%-*cutoff*_)

The diagnostic accuracy of PDA as a function of examination methods and criteria for diagnostic hypotheses sorting is presented in Table 4. The use of the Four Examinations of examination yielded the best diagnostic accuracy and associated statistical performance, followed by three (Ip+AO+Iq), two (Ip+AO) and one method (Ip), despite the use of *N*_% _or *N*_%-*cutoff *_as the secondary ranking criterion.

**Table 4:** Diagnostic accuracy according to the examinations

Methods	Ip		Ip+AO		Ip+AO+Iq		Ip+AO+Iq+P (Four Examinations)	
	
Criterion	N%	N%-52.0%	N%	N%-51.5%	N%	N%-33.5%	N%	N%-28.5%
**N simulated**	13800	13800	13800	13800	13800	13800	13800	13800
N missing*	200	200	200	200	0	0	0	0
**N valid**	13600	13600	13600	13600	13800	13800	13800	13800
**TP**	3033	3349	3502	4021	5791	6085	5951	6199
**FP**	46	40	37	44	25	33	18	35
**FN**	3767	3451	3298	2779	1109	815	949	701
**TN**	6754	6760	6763	6756	6875	6867	6882	6865
**Accuracy**	72.0% (71.2;72.7)	74.3% (73.6;75.1)	75.5% (74.8;76.2)	79.2% (78.6;79.9)	91.8% (91.3;92.3)	93.9% (93.5;94.3)	93.0% (92.6;93.4)	94.7% (94.3;95.1)
**Sensitivity**	44.6% (43.4;45.8)	49.2% (48.1;50.4)	51.5% (50.3;52.7)	59.1% (58.0;60.3)	83.9% (83.1;84.8)	88.2% (87.4;88.9)	86.2% (85.4;87;1)	89.8% (89.1;90.6)
**Specificity**	99.3% (99.1;99.5)	99.4% (99.2;99.6)	99.5% (99.3;99.6)	99.4% (99.2;99.5)	99.6% (99.5;99.8)	99.5% (99.4;99.7)	99.7% (99.6;99.9)	99.5% (99.3;99.7)
**Predictive value (-)**	64.2% (63.3;65.1)	66.2% (65.3;67.1)	67.2% (66.3;68.2)	70.9% (70.0;71.8)	86.1% (85.4;86.9)	89.4% (88.7;90.1)	87.9% (87.2;88.6)	90.7% (90.1;91.4)
**Predictive value (+)**	98.5% (98.1;99.0)	98.8% (98.4;99.2)	99.0% (98.7;99.3)	98,9% (98.6;99.3)	99.6% (99.4;99.8)	99.5% (99.3;99.7)	99.7% (99.6;99.9)	99.4% (99.3;99.7)

Ip: inspection, AO: auscultation-olfaction, Iq: inquiry, P: palpation, TP: true positive, FP: false positive, FN: false negative, TN: true negative, N: number of cases, *N*_%_: quantity of available information

Note: Missing cases are due to absence of manifestations describing the inspection method.

Among all combinations of examination methods tested, there was a statistically significant difference between *N*_% _and *N*_%-*cutoff *_(*P *< 0.001), indicating a better performance of *N*_%-*cutoff *_as a criterion. Higher values of accuracy, sensitivity, specificity (only for Ip), negative predictive value and positive predictive value (only for Ip) were observed when diagnostic hypotheses were ranked by *N*_%-*cutoff *_compared to *N*_%_. However, specificity and positive predictive values were significantly higher (P < 0.001) when diagnostic hypotheses were ranked by *N*_% _for the combinations Ip+AO, Ip+AO+Iq and Ip+AO+Iq+P.

## Discussion

This study evaluated the diagnostic accuracy of PDA with the associated method for estimation of cutoff values for optimization of its accuracy. The findings indicate that early, unbalanced health status may be accurately assessed through pattern differentiation even if little information about the pattern itself is available. The computational strategies implemented provided statistical data related to the Chinese medicine therapeutic principle of treating who is not yet ill.

### Assessment of early unbalanced health status

The concave shape (Figure 4) of accuracy curves can be explained by the following reasons. As *N*_% _tends toward zero, the low average diagnostic accuracy resulted from the selection of manifestations that collectively did not represent a unique description of any pattern on the dataset. Likewise, as *N*_% _tends toward 100%, the low average diagnostic accuracy resulted from the selection of too many manifestations that generate several unique subgroups, including more candidate patterns (observed increase in the number of candidate patterns in Figure 5).

Regarding *Zang-fu *single patterns, those results suggest that PDA is more likely to perform successful pattern differentiation if about 28.5% of the information of the actual pattern is available using the Four Examinations. It is still possible to obtain an accurate diagnosis with lower or higher values of available information (Figure 3), but at lower expected frequencies. Moreover, the results do not indicate that any subset with 28.5% of the manifestations allow an accurate *Zang-fu *pattern differentiation because all manifestations were chosen at random. The discovery of which subsets lead to a more accurate diagnosis needs further evaluation. Methods such as latent tree models [34] may be used to identify such subsets. Finally, the concomitant increase in statistical performance and decrease in *N*_% _as a function of combined exam methods reinforces the Four Examinations as the best practice for *Zang-fu *single pattern differentiation when little information is available.

PDA was implemented to differentiate the correct pattern among possible ones rather than to identify the healthy/sickness outcome [14]. In the current version of PDA, it is not possible to obtain the healthy status. However, a possible outcome is that no pattern satisfactorily explains the manifestations presented by the patient (non-unique, higher *F*_% _and non-unique, lower *N*_%_). Such a case may need further exploration by the Chinese medicine practitioner to determine if the patient is actually sick. For PDA to obtain the "healthy pattern," it must be described in the same manner as the patterns already in the dataset. For instance, the "liver-blood deficiency" *Zang-fu *pattern should have the mutually exclusive counterpart "liver-blood health" pattern. The description of such health-related patterns is possible because Chinese medicine is not limited to assessing presence/absence of manifestations (e.g. asymptomatic individuals may be also diagnosed). Constitutional and behavioral aspects are also considered relevant to establish the true pattern [9]. A completely healthy person will present all healthy patterns simultaneously. However, in clinical practice it is difficult to find a patient that is completely healthy and thus the pattern differentiation process must be applied. Future works should incorporate these aspects to improve PDA's validity and clinical use as a screening test between health and disease.

### To treat who is not yet ill

The proposed computational strategies are based on the explained (*F*_%_) and available (*N*_%-*cutoff*_) information. Both strategies preserve the traditional concepts of patterns. The quantity of explained information, *F*_%_, allows the interpretation of manifestations collectively, that is, diagnostic hypotheses are ranked by the strength of the simultaneous occurrence of manifestations rather than individual manifestations. Additionally, *N*_%-*cutoff *_is based on the hypothesis of optimum information for diagnosis, which was confirmed in this study at least for *Zang-fu *single patterns. Thus, when *F*_% _is identical among diagnostic hypotheses, the optimized available information (measured with *N*_%-*cutoff*_) can be used to rank patterns to better identify the diagnosis.

The significant increase in diagnostic performance (accuracy, sensitivity, and negative predictive value) using *N*_%-*cutoff *_compared to *N*_% _reflect the recognition of more true positive cases with this secondary criterion. The significant decrease in performance (specificity and positive predictive value) occurred because PDA identified an incorrect diagnosis in true negative cases using *N*_%-*cutoff *_as the decision criterion when two or more patterns presented the same *F*_% _(when no diagnosis would be identified by *F*_% _alone).

In the theoretical field, this study provides statistical data related to one of the most important Chinese medicine principles, namely "treat who is not yet ill" [4-6,25]. Patterns may be transmitted among *Zang-fu *by fixed or not fixed laws [9,25], adding manifestations or changing their severity and location. However, *Zang-fu *patterns in early stages may be well explained by a single pattern, as it represents the first stage of progression through the *Zang-fu *system [9]. Thus, patterns must be recognized in early stages so treatment of unaffected systems can be initiated. As such, the ability to detect single patterns is of extreme relevance if the treatment is intended to be immediately started. PDA correctly identified 94.7% of the cases as true positive or true negative and can be used to help Chinese medicine practitioners in the pattern identification process of *Zang-fu *single patterns.

### Simulated manifestation profiles versus real cases

Studies on the evaluation of accuracy of Chinese medicine diagnostic methods [1,3,12] relied on real cases instead of simulated cases. Those studies used the diagnoses given by Chinese medicine practitioners as the gold-standard, which may not be reliable because of the low agreement among practitioners [34]. Simulation of manifestation profiles is more appropriate for diagnostic accuracy studies because it guarantees that the diagnosis is known. Moreover, the implemented stochastic method allows a focus on the properties of manifestation profiles instead of individual manifestations [23]. This procedure is to generate a large number of examples of any given pattern (stochastic process) and then examines the relative proportion of successes of the diagnostic test (deterministic process) [24]. The proposed MPSA is suitable for diagnostic studies because it tests several cases found in clinical practice. The main advantage of the simulation method is that the actual diagnosis is known from the dataset, which means that no gold-standard method is necessary.

Epidemiological information for manifestations describing *Zang-fu *(or any other theory-based) patterns is insufficient in the current literature [35]. Studies with small sample sizes [36-39] described the frequencies of manifestations among patterns but were not designed to obtain these data in the general population. Moreover, there is no description of mathematical models to simulate frequency distributions of the *Zang-fu *manifestations. Any assumed distribution may introduce selection bias (by increasing the frequency of low-probability manifestations and/or decreasing the frequency of high-probability ones). Future clinical research should focus on determining the distribution of manifestations in each pattern (and patterns in the dataset) to further improve the MPSA.

Another possible influence is the dataset which comprises the core of the process. Different datasets may result in better or worse diagnostic accuracies (as well as cutoff values) depending on characteristics such as the quantity of patterns and manifestations, use of specific or general terms to describe the manifestations and co-occurrence of manifestations among single patterns. Those datasets should be arranged by the Four Examinations so that the same evaluation can be made and comparisons performed. Such comparisons would evaluate the use of PDA for clinical and research purposes. This aspect stresses the need for internationally available Web-based datasets [3].

MPSA and PDA are related to single patterns, which is the simplest case found in clinical practice. In fact, a patient may present several patterns simultaneously that are not mutually exclusive (i.e. lung-qi deficiency and spleen-qi deficiency), which may explain the decrease of accuracy when more information was made available. Additionally, common etiologies and pattern transmissions among *Zang-fu *- which is also implied in the determination of the Root-Manifestation relationship - are factors that must be incorporated into MPSA and PDA to extend its application in general clinical practice for detection of *Zang-fu *single and multiple patterns.

### PDA compared to other computational models

Comparative analyses could not be performed for the following reasons. Datasets frequently used to test classification algorithms are available at the UCI repository http://www.ics.uci.edu, including the results recently reported by Wang *et al*. [40] using the SPECT Heart Dataset, Lung Cancer Dataset, and Iris Dataset. However, there is not a dataset in this repository with a compatible format to test PDA's performance. Many of the datasets are composed by categorical variable (the type used by PDA) and other variables types. It would not be a valid comparison simply to ignore the real or ordinal variables type because they provide relevant information for classification of those datasets. Even if those variables were ignored, no dataset presents a wide description of Chinese medicine patterns (only real or simulated cases). Although it is possible to apply a "reverse model" and recreate the dataset from these presented cases (by grouping all cases from the same classification pattern and removing repetitive data), the attributes distributed with this procedure would not cover the Four Examinations. Finally, if the dataset were constructed and the cases from this repository were tested regardless of the issues stated above, they do not present the variable of amount of information and thus no cutoff value for this variable could be obtained to test its influence on the PDA's accuracy.

### Implications for research and clinical use

Despite the lack of comparison with other methods, some inferences can be made by comparing PDA with other diagnostic methods. PDA has the several major advantages over the conventional learning algorithms. There is no need to train PDA, which is a mathematical process already subject to bias. PDA's method is simple, and both criteria (*F*_% _and *N*_%_) can be calculated even manually by a Chinese medicine practitioner (for a low number of candidate patterns). Its reasoning is entirely based on the actual process executed by Chinese medicine experts [4-8] and thus reduces the error in data collection and analysis. The practitioner can add, change or delete manifestations in the dataset based on other Chinese medicine publications and then retest the impact of those changes in terms of diagnostic accuracy with the methods describe in this work. Finally, PDA is more stable than other learning algorithms because the final diagnosis does not depend on the initial guess (or sequence of manifestations) used during the learning phase (i.e. given the same manifestations PDA will always provide the same diagnosis, no matter the sequence in which the manifestations are collected).

Recent studies on Chinese medicine have focused on computer aids to diagnose patients [1,3,12,40,41], prescribe treatment [2] and statistical methods [13,14] for validation of Chinese medicine theories. It is believed that the combination of traditional theories and modern techniques may improve the scientific approach of Chinese medicine interventions by means of computerization and standardization of diagnosis and treatment based on traditional rules. Practitioners may implement algorithms to become "superior doctors." In this direction, PDA can be used by Chinese medicine practitioners and researchers as a software tool for real-time assessment of patients with actual information about its statistical performance.

## Conclusion

This study suggests that pattern differentiation based on both explained and optimum available information (*F*_% _and *N*_%-*cutoff*_) is more accurate than using explained and available information without a cutoff (*F*_% _and *N*_%_). Both *F*_% _and *N*_%-*cutoff *_should be used as PDA's objective criteria to perform *Zang-fu *single pattern differentiation.

## Abbreviations

Ip: inspection; AO: auscultation and olfaction; Iq: inquiry; P: palpation; PDA: pattern differentiation algorithm; ROC: receiver operating characteristic;*K*: single pattern from dataset; MPSA: manifestation profile simulation algorithm; *N*_*T*, *K*_: quantity of manifestations describing pattern *K *in dataset; *AUC*: area under the curve; *N*_*R*, *K*_: quantity of randomly selected manifestations of pattern *K*; *N*_%, *K*_: proportion of available information of pattern *K *in dataset; *F*_%, *K*_: proportion of explained information of pattern *K *from clinical history; *N*_%-*cutoff*_: proportion of optimized available information of pattern *K *in dataset; *N*_*E*, *K*_: quantity of explained manifestations of pattern *K*; *N*_*P*_: quantity of presented manifestations on the clinical history; *N*_*CP*_: quantity of candidate patterns; 95%*CI*: 95% confidence interval; *p*: binomial proportions; *N*_%, *S*_: quantity of cases at *N*_% _with successful procedure; *N*_%, *U*_: quantity of cases at *N*_% _with unsuccessful procedure; *n*_S_: sample size with successful procedure; *n*_*U*_: sample size with unsuccessful procedure; *Q*_1_: probability that two randomly chosen unsuccessful procedures will both be ranked with greater suspicion than a randomly chosen successful procedure; *Q*_2_: probability that one randomly chosen unsuccessful procedure will be ranked with greater suspicion than two randomly chosen successful procedures

## Competing interests

The author declares that they have no competing interests.

## Authors' contributions

The author performed the study; wrote, revised and approved the manuscript.

## Supplementary Material

Additional file 1 **Sixty-nine (69) *Zang-fu *single patterns described in the dataset**. This table lists the 69 *Zang-fu *single patterns described in the dataset. Click here for file
