# Supplementary material for: Diagnostic accuracy of pattern differentiation algorithm based on Chinese medicine theory: a stochastic simulation study
Source: Chin Med. 2009 Dec 21;4:24. doi: 10.1186/1749-8546-4-24 (PMC2806360; doi:10.1186/1749-8546-4-24)
Supplement: Additional file 1 — Sixty-nine (69) Zang-fu single patterns described in the dataset. This table lists the 69 Zang-fu single patterns described in the dataset. [file 1749-8546-4-24-S1.DOC]

# Sixty-nine (69) *Zang-fu* single patterns described in the dataset

| 1. Deficiency-cold in the bladder | 36. Phlegm-fluids obstructing the lungs |
| --- | --- |
| 2. Kidney-qi deficiency | 37. Heart-yang deficiency |
| 3. Cold invading the large intestine | 38. Phlegm-heat obstructing the lungs |
| 4. Large intestine dryness | 39. Heart-yin deficiency |
| 5. Cold invading the stomach | 40. Retention of food in the stomach |
| 6. Liver-blood deficiency | 41. Heat in the large intestine |
| 7. Collapse of the large intestine | 42. Deficiency-cold in the small intestine |
| 8. Liver-blood stasis | 43. Heat obstructing the large intestine |
| 9. Damp-cold in the bladder | 44. Small intestine qi pain |
| 10. Liver-fire blazing upwards | 45. Infestations of worms in the small intestine |
| 11. Damp-cold invading the spleen | 46. Small intestine qi tied |
| 12. Liver-qi stagnation | 47. Invasion of the large intestine by cold |
| 13. Damp-heat in the liver and gall bladder | 48. Spleen not controlling the blood |
| 14. Liver wind agitating within, deficient liver-blood causing wind | 49. Invasion of the lungs by wind-cold |
| 15. Damp-heat in the bladder | 50. Spleen-qi deficiency |
| 16. Liver wind agitating within, extreme heat generating wind | 51. Invasion of the lungs by wind-heat |
| 17. Damp-heat in the gall bladder | 52. Spleen-qi sinking |
| 18. Liver wind agitating within, liver-yang rising causing wind | 53. Invasion of the lungs by wind-water |
| 19. Damp-heat in the large intestine | 54. Spleen-yang deficiency |
| 20. Liver-yang rising | 55. Kidney-essence deficiency |
| 21. Damp-heat invading the spleen | 56. Stagnation of cold in the liver channel |
| 22. Liver-yin deficiency | 57. Kidney-qi not firm |
| 23. Full-heat in the small intestine | 58. Stasis of blood in the stomach |
| 24. Lung dryness | 59. Kidneys failing to receive qi |
| 25. Gall bladder deficiency | 60. Deficiency-cold in the stomach |
| 26. Lung-qi deficiency | 61. Kidney-yang deficiency |
| 27. Heart-blood deficiency | 62. Stomach-fire |
| 28. Lung-yin deficiency | 63. Kidney-yang deficiency, water overflowing to the heart |
| 29. Heart-blood stasis | 64. Stomach-qi deficiency |
| 30. Phlegm misting the mind | 65. Kidney-yang deficiency, water overflowing to the lungs |
| 31. Heart-fire blazing | 66. Stomach qi rebelling upwards |
| 32. Phlegm-damp obstructing the lungs | 67. Kidney-yin deficiency |
| 33. Heart-qi deficiency | 68. Stomach-yin deficiency |
| 34. Phlegm-fire harassing the heart | 69. Kidney-yin deficiency, empty-fire |
| 35. Heart-yang collapse |  |
